# Comparison of Accelerated and Standard Corneal Collagen Cross-Linking Treatments in Experimental Fungal Keratitis for *Aspergillus fumigatus*

**DOI:** 10.1155/2022/1085692

**Published:** 2022-07-20

**Authors:** Anji Wei, Zhennan Zhao, Xiangmei Kong, Tingting Shao

**Affiliations:** ^1^Department of Ophthalmology and Vision Science, Eye Ear Nose and Throat Hospital of Fudan University, Shanghai, China; ^2^NHC Key Laboratory of Myopia (Fudan University), Laboratory of Myopia, Chinese Academy of Medical Sciences, Shanghai, China; ^3^Key Laboratory of Visual Impairment and Restoration of Shanghai, Shanghai, China; ^4^Department of Ophthalmology, The First Affiliated Hospital of Nanchang University, Nanchang, China

## Abstract

**Introduction:**

To compare accelerated and standard corneal collagen cross-linking (CXL) treatments in experimental *Aspergillus* keratitis models.

**Methods:**

Twenty-six New Zealand rabbits were divided into two groups: a 1% voriconazole combined with standard CXL group, and a 1% voriconazole combined with accelerated CXL group. The ulcer area, corneal opacity, and corneal neovascularization score were measured via slit-lamp imaging, and the corneal and corneal epithelial thickness and ulcer depth were measured via anterior segment optical coherence tomography (AS-OCT). The duration of the hyphae was observed via *in vivo* confocal microscopy (IVCM), and the cornea was taken for pathological examination after modeling and at the end of the study to determine the hyphae and corneal repair. The observation times were as follows: at successful modeling (day 0) and at 1, 4, 7, 14, 21, and 28 days after the intervention.

**Results:**

The area and depth of the ulcer decreased in both groups after CXL (all *P* < 0.05). Interestingly, the ulcer area in the accelerated CXL group still tended to increase on the first day after CXL although the difference was not statistically significant (*P*=0.6649). On the 21st and 28th days after CXL, the ulcer area and depth of the standard CXL group were larger and deeper than those of the accelerated CXL group (all *P* < 0.05). The ulcer healing time in the accelerated CXL group was 18.67 ± 6.21 days, while that in the standard CXL group was 23.55 ± 4.72 days, and the difference was statistically significant (*P*=0.0475).

**Conclusions:**

Both accelerated and standard CXL can significantly inhibit the progression of *Aspergillus* keratitis corneal ulcers and promote ulcer healing. The accelerated CXL was superior to the standard CXL, which could control infection faster and promote ulcer healing. However, it is important to note that there may be a risk of early deterioration of the ulcer with accelerated CXL.

## 1. Introduction

Corneal collagen cross-linking (CXL) is a therapeutic method that mediates the cross-linking reaction of collagen fibers by using riboflavin as a photosensitized agent and ultraviolet (UV) irradiation to improve the biomechanical properties of the cornea [1]. It is mainly used for the treatment of ectatic diseases, such as keratoconus [1, 2]. The Dresden protocol was used for the standard CXL. Its UV setting parameters were wavelength (365 ± 5) nm, total energy 5.4 J/cm^2^, irradiation intensity 3.0 mW/cm^2^, irradiation time 30 minutes, 0.1% riboflavin drops continuously for 30 minutes before irradiation, and a required time of more than 1 hour [1]. The patient has to stay in the same position for more than an hour, which is a challenge for both the surgeon and the patient. According to the Bunsen-Roscoe law [3], the total energy of light absorbed in photochemical effects is proportional to the product of the irradiation time and the intensity of light. Therefore, some scholars try to increase the irradiation intensity to reduce the irradiation time, so as to achieve the purpose of keeping the total irradiation energy unchanged, that is, the accelerated CXL [4, 5]. Animal and clinical studies have shown that the corneal biomechanics change, as the cross-linking of these protocols is similar to those of standard CXL protocols without significant side effects [6–10]. Currently, the commonly used accelerated CXL parameter is 9 mW/cm^2^. However, these attempts only focus on how to effectively and safely improve the post-CXL corneal biomechanics, rather than its anti-infection ability [5, 10].

Fungal keratitis is a serious infectious corneal disease with a high rate of blindness [11–13]. For fungal keratitis, existing drugs mostly have low penetration, many side effects, and increasing drug resistance [14]. Penetrating keratoplasty is often required in severe cases, but the lack of donor material makes treatment difficult [12, 15, 16]. Since Iseli et al. reported 5 cases of fungal and bacterial infections successfully treated by CXL [17], scholars have increasingly applied it in the treatment of infectious corneal ulcers, and different CXL protocols have achieved good efficacy in both in vitro and in vivo studies [18–25]. Our previous study confirmed that the treatment effect of standard CXL combined with voriconazole was significantly better than that of voriconazole alone in a rabbit model of *Aspergillus* infected corneal ulcers.

At present, no systematic study has compared the therapeutic effects of CXL with different parameter settings on fungal keratitis. Therefore, based on our previous animal model research, standard CXL (3 mW/cm^2^*∗* 30 min) and accelerated CXL (9 mW/cm^2^*∗* 10 min) were selected for this study.

## 2. Materials and Methods

### 2.1. *Aspergillus fumigatus*

*The Aspergillus fumigatus* was provided by The Department of Infectious Diseases, Huashan Hospital, affiliated with Fudan University. The *Aspergillus fumigatus* was cultured on potato dextrose agar (PDA) at 25°C for 7 days, and the cultures were picked and diluted to 0.5 McFarland standard units with sterile saline under a McFarland turbidimeter, equivalent to 10^8^ CFU/mL (colony forming units/ml). The actual concentration was determined by the colony counting method and diluted to 10^6^ CFU/mL according to the ratio of 1 : 100.

### 2.2. *Aspergillus fumigatus* Infection Model in Rabbit

Twenty-six specific pathogen-free (SPF) male New Zealand white rabbits with a body weight of 2.5∼3.0 kg were selected and provided by the Department of Pediatrics, Medical College of Fudan University (Shanghai, China). All the experimental methods used in this study were in accordance with the Helsinki Declaration, and the animal experiments were conducted in accordance with the regulations of the Society of Vision and Ophthalmology (ARVO) on the use of experimental animals in ophthalmic research and approved by the Animal Ethics Committee of Fudan University Medical School (Shanghai, China).

All rabbits were treated with an intramuscular injection of 35 mg/kg of intramuscular ketamine hydrochloride and 5 mg/kg of xylazine for general anesthesia, and ambucaine hydrochloride eye drops were used for local anesthesia (Santen Pharmaceutical Co., Ltd.). Then, 50 *μ*L *Aspergillus fumigatus* suspension (10^6^ CFU/mL) was extracted, and corneal stroma injection was performed at the depth of 1/3∼2/3 total corneal thickness. Seventy-two hours after the injection, typical ulcers (corneal infiltrate associated with an overlying epithelial defect) were formed, and hyphae were detected by *in vivo* confocal microscopy (IVCM; HRT3-RCM, Heidelberg Engineering, GmbH, Heidelberg, Germany), which was determined to be *Aspergillus fumigatus* infection. It was recorded as day 0 of the experiment, and experimental observation was started. At the same time, one experimental rabbit was sacrificed in each group (see grouping below), and corneal tissue was taken for pathological sections to determine the presence of hyphae.

### 2.3. Experimental Animal Grouping and Follow-Up

The remaining 24 rabbits were randomly divided into two groups: group A (accelerated CXL group, CXL-A; *n* = 12, 9 mW/cm^2^*∗*10 min) and group B (standard CXL group, CXL; *n* = 12, 3 mW/cm^2^*∗*30 min). After CXL, both groups were treated with 1% voriconazole (Vfend IV, Pfizer Pharmaceuticals, New York, USA) eye drops, 3 h/time, 4 times/day. Data were collected at 0, 1, 4, 7, 14, 21, and 28 days after the intervention. In addition, before modeling, all experimental rabbits underwent anterior segment optical coherence tomography (AS-OCT; RTVue Version 6.9 Optovue Inc., Fremont, CA, USA) scanning to record corneal and corneal epithelial thicknesses. On day 28, the rabbits in each group were sacrificed after data collection, and the corneal tissues of the eyes were collected for pathological analysis.

### 2.4. Corneal Collagen Cross-Linking

The corneal epithelium and necrotic tissue (9 mm in diameter, including the ulcer area) in the central area were removed, and 0.1% riboflavin (Medio-Cross riboflavin/dextran solution) was applied to the eye for every three minutes for 30 minutes. Cross-linking was performed using the KXL System (Avedro, Waltham, Mass., USA). In group A, the UV irradiation parameters were set at 9 mW/cm^2^, and the cross-linking time was 10 minutes. The UV irradiation parameters of group B were set at 3 mW/cm^2^, and the cross-linking time was 30 minutes. In both groups, cross irradiation was placed in the center of the ulcer with a diameter of 11 mm, and 0.1% riboflavin was added every 5 minutes.

### 2.5. Ophthalmologic Examinations

All rabbits underwent the following eye examinations: slit-lamp imaging, AS-OCT scans, and IVCM. Slit-lamp imaging were performed with uniform magnification and scale, and the areas of the corneal ulcers were analyzed using ImageJ software. Corneal tomography was obtained using AS-OCT scans, and the following parameters were measured: maximum ulcer depth, corneal thickness after ulcer healing, and corneal epithelial thickness. The healing of the corneal ulcers was deﬁned as complete reepithelialization and resolution of inﬁltrate and hypopyon. The hypha, spores, and ulcer development of *Aspergillus fumigatus* in and around the corneal ulcer area were observed by IVCM. Corneal opacity and corneal neovascularization were scored according to the Schreiber scoring system [26].

### 2.6. Pathological Observation of Corneal Tissue in Rabbit

After the modeling and the experiment, the corneal tissue was collected. Hematoxylin–eosin (HE) staining was used for the histopathological analysis of all groups. Corneal tissue was fixed with 4% neutral formaldehyde solution, dehydrated, embedded in paraffin, and sectioned continuously with a thickness of 3–4 *μ*m. Bake the slices for 1 hour at 60°C, dyed with HE, transparent with xylene, and sealed with neutral gum. The ImageJ software was used to analyze the inflammatory cell density.

### 2.7. Statistical Analysis

The Statistical Package for the Social Sciences (SPSS) 23.0 software was used for the data analysis. The ulcer area and depth, corneal thickness, and corneal epithelial thickness between the groups were compared using one-way analysis of variance (ANOVA). The ulcer area and depth within the group were compared using repeated measures ANOVA. The corneal opacity score and corneal neovascularization score between the two groups were compared using the independent sample Kruskal-Wallis test. The healing rates of the two groups were compared using Fisher's exact test. Descriptive statistics were expressed as mean ± standard deviation (SD), and *P* < 0.05 was regarded as a statistically significant difference.

## 3. Results

**Figure 1:**
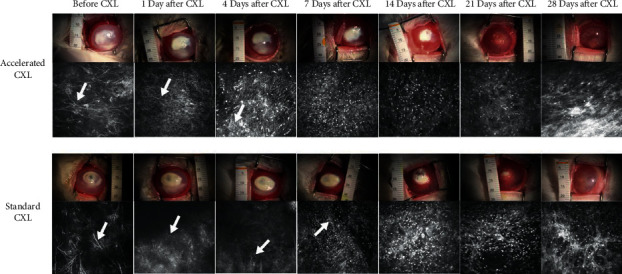
Representative images of the corneal ulcer of a representative rabbit and its corresponding IVCM in each group at all follow-up periods. The white arrow shows the fungal hypha. CXL, standard corneal collagen cross-linking.

**Figure 2:**
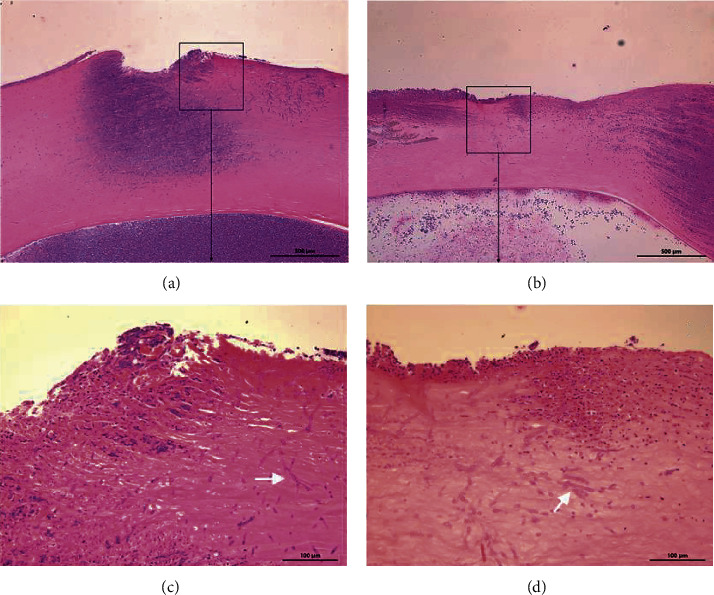
Histopathology of corneas in each group 3 days after corneal stroma injection. The corneal tissue of rabbit in each group was subject to HE staining. Figure (a) and (c) accelerated CXL group; Figure (b) and (d) standard CXL group. Figure (c) and (d) (X20) are the magnification of the content of the boxes in group (a) and (b) (X5), respectively; and the white arrow refers to the fungal hyphae.

Three days after the corneal stromal injection, typical corneal ulcers were formed in all rabbits. Corneal stromal hyphae were seen in the IVCM (Figure 1) and pathological sections (Figure 2). The model of the rabbits' *Aspergillus* corneal ulcers was successful. Twelve rabbits in each group were included in the final analysis.

**Figure 3:**
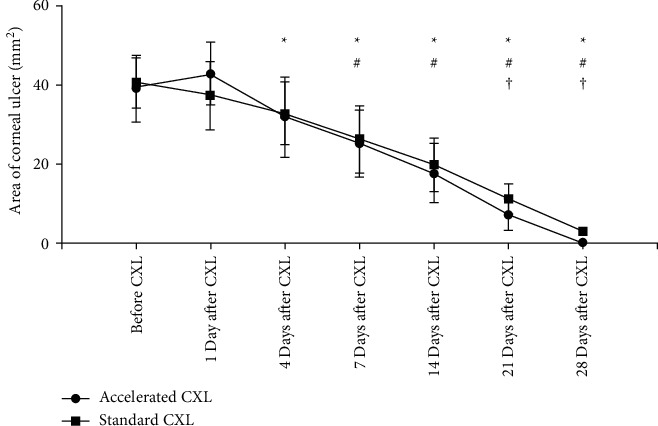
The area of corneal ulcer in two groups. ^*∗*^ and ^#^ The corneal ulcer area was significantly different at different follow-up time point in each group compared with that before treatment (^*∗*^standard CXL group, #accelerated CXL group; *P* < 0.05 for all). ^†^ There were significant differences in the areas of corneal ulcer between each group at one follow-up time point (*P* < 0.05 for all). CXL, corneal collagen cross-linking.

The changes in the ulcer areas among and between the two groups are shown in Figure 3. After CXL, the standard CXL group showed a decreasing trend from the first day, but the difference was not statistically significant (*P*=0.7038). Ulcer area decreased from day 4 to day 28 after CXL (all *P* < 0.05). Interestingly, the ulcers in the accelerated CXL group still tended to increase on the first day after CXL although the difference was not statistically significant (*P*=0.6649). Subsequently, the ulcer areas decreased as compared with before, and from the 7th day after CXL, the ulcer areas decreased with statistical significance (all *P* < 0.0001).

The initial ulcer areas of the standard CXL group and the accelerated CXL group were 40.53 ± 6.37 mm^2^ and 39.15 ± 8.52 mm^2^, respectively; and there was no statistical difference (*P*=0.6576). The comparison of the ulcer areas between the two groups at the same time point after CXL showed that there was no statistical significance in ulcer area between the two groups from the first day to the 14th day after surgery (all *P* > 0.05). On days 21 and 28, the ulcer areas in the standard CXL group were larger than those in the accelerated CXL group, with the difference being statistically significant (*P*=0.0188, *P* < 0.0001). It is suggested that the ulcer reduction of the accelerated CXL was better than that of the standard CXL group on the 21st and 28th days after CXL. The ulcer changes in the two groups at different times are represented in Figure 3.

**Figure 4:**
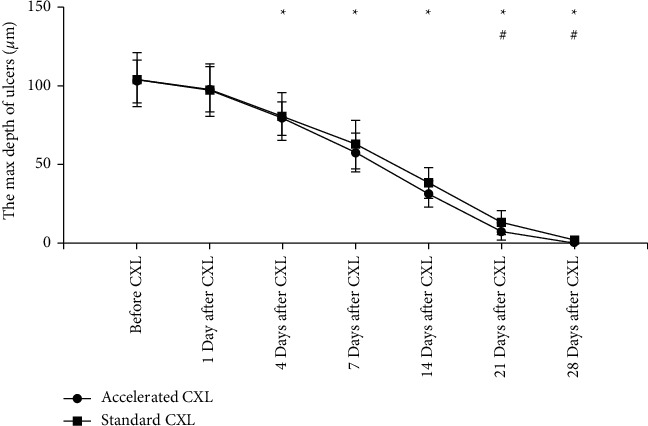
The depth of corneal ulcer in two groups. ^*∗*^ The corneal ulcer depth was significantly different at different follow-up time point in each group compared with that before treatment (*P* < 0.05 for all). ^#^There were significant differences in the depth of corneal ulcer between each group at one follow-up time point (*P* < 0.05 for all). CXL, corneal collagen cross-linking.

Similarly, the changes in ulcer depth between the two groups are shown in Figure 4. On the first day after CXL, the ulcer depth in both groups became shallower, but the difference was not statistically significant (*P*=0.6408, *P*=0.7233). From the fourth day after CXL to the end of the experiment, ulcer depth continued to become shallower in both groups, with statistical significance (all *P* < 0.05). The initial ulcer depths of the standard CXL group and the accelerated CXL group were 103.90 ± 13.53 *μ*m and 102.76 ± 8.52 *μ*m, respectively, with no statistical difference (*P*=0.8611). After CXL, the trend of shallower ulcer depths was similar between the two groups. On the 21st and 28th day after CXL, the ulcer depth of the standard CXL group was deeper than that of the accelerated CXL group, and the difference was statistically significant (*P*=0.048, *P* < 0.0001).

The accelerated CXL group achieved epithelial healing at the end of the study; while one rabbit in the standard CXL group had incomplete ulcer healing, and there was no difference in the ulcer healing rate between the two groups. The ulcer healing time in the accelerated CXL group was 18.67 ± 6.21 days, while that in the standard CXL group was 23.55 ± 4.72 days, and the difference was statistically significant (*P*=0.0475). The IVCM showed that the hyphae existed for 3.33 ± 2.50 days in the accelerated CXL group and 5.67 ± 2.18 days in the standard CXL group, and the difference was not statistically significant (*P*=0.0509).

The corneal and corneal epithelial thicknesses between the two groups before modeling and at the end of the experiment are shown in Table 1. It can be seen that there was no difference in the thickness of the cornea and corneal epithelium between the two groups before modeling. At the end of the experiment, the cornea and corneal epithelium in both groups were thicker than before modeling (all *P* < 0.05), but there was no significant difference between the two groups. At the end of the experiment, the corneal opacity score between the two groups was 2.42 ± 0.51 (accelerated CXL group) and 2.50 ± 0.52 (standard CXL group), respectively, and the difference was not statistically significant (*P* > 0.999). The corneal neovascularization score between the two groups was 2.67 ± 0.98 (accelerated CXL group) and 3.16 ± 0.72 (standard CXL group), and the difference was not statistically significant (*P*=0.2256).

**Figure 5:**
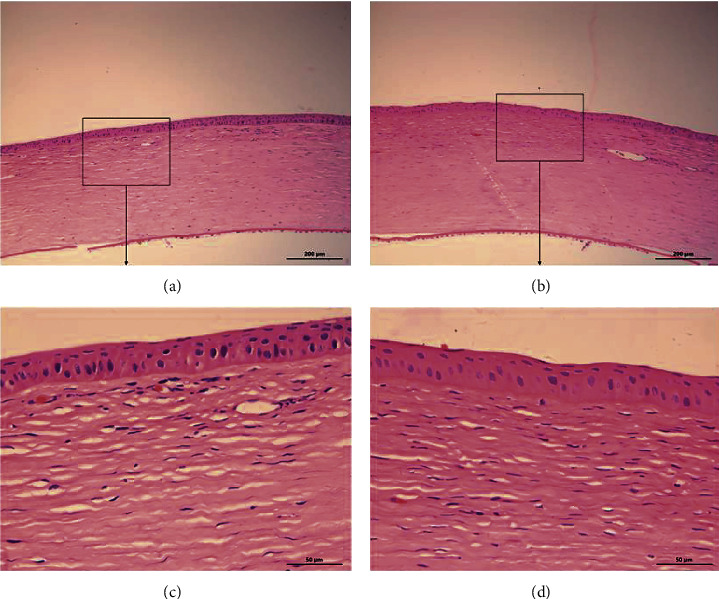
Histopathology of corneas in each group 28 days after CXL. The corneal tissue of rabbit in each group was subject to HE staining. Figure (a) and (c) accelerated CXL group; Figure (b) and (d) standard CXL group. Figure (c) and (d) (X40) are the magnification of the content of the boxes in figure (a) and (b) (X10), respectively.

The HE-stained sections of the two groups (Figure 5) were observed under a light microscope 28 days after CXL, and the inflammatory cells were counted. The typical results were as follows: the corneal ulcers had healed, the corneal epithelium was flattened, the corneal stromal fibroblasts proliferated, the fibrous tissues were regularly arranged, neovascularization could be seen locally, there was inflammatory cell infiltration, and Descemet's membrane and endothelial layer were generally normal. Further comparison showed that the distribution of the corneal stromal dense layer and new blood vessels after CXL in the accelerated CXL group was more superficial than that in the standard CXL group; the density of corneal stromal inflammatory cells between the two groups was 51.28 ± 9.56 cell/mm^2^ (accelerated CXL group) and 59.74 ± 12.22 cells/mm^2^ (standard CXL group), and the difference was not statistically significant (*P*=0.072).

## 4. Discussion

This study compared the effects of accelerated CXL and standard CXL combined with voriconazole on *Aspergillus* infected corneal ulcer in rabbits. Both protocols were able to significantly inhibit the progression of *Aspergillus* keratitis corneal ulcers and promote ulcer healing. Notably, accelerated CXL combined with voriconazole showed a stronger advantage in ulcer healing.

In terms of the overall change in ulcer areas and ulcer depths in the two groups, the similarity trend of corneal ulcer areas and ulcer depths between the two groups was inhibited after the two protocols of CXL treatment. By the end of the experiment, most of the ulcers in the two groups had healed, and there was no difference in the ulcer healing rate between the two groups. The principles of CXL in the treatment of infectious keratopathy include eliminating pathogenic microorganisms, increasing corneal biological stability and antienzyme activity, and reducing associated inflammatory responses [21, 27, 28]. In animal models of Fusarium and Candida albicans infection, both standard and accelerated CXL protocols have been shown to be effective [28, 29]. In *in vitro* culture experiments, Bilgihan et al. confirmed the bacteriostatic effect of the accelerated cross-linking protocol on *Aspergillus* [19]. Our previous study also confirmed that the effect of standard CXL combined with voriconazole on *Aspergillus* infected rabbit corneal ulcers was significantly better than that of voriconazole alone.

In this study, although the irradiation intensities of the two groups of CXL protocols were different (3 mW/cm^2^ and 9 mW/cm^2^), the total energy of irradiation was the same at 5.4 J/cm^2^. Previous experiments have confirmed that the schemes with the same total energy had the same biomechanical changes after cross-linking; that is, the mechanical strength and stability of the cornea, as well as the degree of improvement in enzymatic resistance, were similar [6–9]. Richoz et al. found that the bactericidal effects of 36 mW/cm^2^ irradiation for 2.5 minutes and 18 mW/cm^2^ irradiation for 5 minutes were basically the same for *Staphylococcus aureus* and *Pseudomonas aeruginosa in vitro* [30]. Currently, a comparative study of different CXL protocols on different fungus-infected corneal ulcers has not been reported. This study is the first one to compare the efficacy of accelerated CXL and standard CXL in *Aspergillus* rabbit corneal ulcers, with the former showing efficacy comparable to standard CXL.

In this study, the ulcer healing time in the accelerated CXL group was shorter than that in the standard CXL group. By day 21, the control of both ulcer areas and ulcer depths was better in the accelerated CXL group than in the standard CXL group. In addition, the existence time of hyphae in the accelerated CXL group was shorter than that in the standard CXL group although the difference was not statistically significant. These results suggest that voriconazole combined with accelerated CXL may be superior to standard CXL in promoting ulcer healing and antifungal ability. According to the Bunsen-Roscoe law [3], the total energy of light absorbed in a photochemical effect is proportional to the product of the irradiation time and the intensity of light. Although the total energy of the two CXL protocols adopted in this study is the same, the response of cells and tissues in the body to electromagnetic radiation often involves a series of complex biological reactions and interferences, resulting in an original linear relationship that is sometimes not fully established. Herekar et al. reported that the oxygen in the reaction zone could be rapidly consumed during the cross-linking process and that the oxygen consumption was proportional to the intensity of UV light [31]. The ultraviolet irradiation intensity of accelerated CXL was significantly higher than that of standard CXL, and the hypoxia state in the cross-linking reaction area may be more unfavorable for the survival and reproduction of fungi. Interestingly, as seen in the HE-stained sections, the residual anterior corneal dense layer of the corneal stroma after cross-linking at the end of this experiment was relatively thin in the accelerated CXL group. Holopainen et al. found that, during the cross-linking process, the corneal thickness was significantly thinner (up to 200 *μ*m locally) due to moisture evaporation [32]. Therefore, we hypothesized that the operation of standard CXL for up to 1 hour was sufficient to cause a more pronounced decrease in intraoperative corneal thickness. Such changes in corneal thickness may lead to differences in cross-linking depth between the two groups. Although cross-linking is effective for fungal corneal ulcers, it can also reduce drug permeability in corneas [33, 34]. However, the relatively deeper cross-linking depth of standard CXL group reduced the penetration of voriconazole in the corneal stroma [33], which may also be the reason why the antifungal ability and healing speed of the standard CXL group were not as good as the accelerated CXL group. The dose-time-effect relationship of cross-linking reactions in infectious corneal diseases has rarely been studied, and further studies are needed to elucidate it.

We also observed a tendency of ulcer enlargement on day 1 after intervention in the accelerated CXL group (cross-linking with higher radiation intensity) compared with before intervention, suggesting that cross-linking with higher radiation intensity (9 mW/cm^2^) may pose a risk of expanding ulcers in the early stage. This may be related to delayed stroma cell apoptosis, which occurred 24 hours after CXL. Studies have shown that the degree of apoptosis is related to the level of UV energy [35], while the accelerated CXL group was cross-linked with higher intensity ultraviolet radiation. Secondly, phototoxic effects may also be involved [36]. In addition, cross-linking may change the antigenic sites of proteins in the corneal stroma, leading to an immune response [36]. At the same time, CXL may also exacerbate the imbalance between the metalloproteinases produced by fungal infection and its tissue inhibitors [37], both of which may contribute to the tendency of an ulcer to expand on the first day after accelerated CXL.

The corneal opacity and corneal neovascularization scores showed no difference between the two groups at 28 days after CXL. At the same time, although there was still inflammatory cell infiltration in the corneal stroma between the two groups, there was no difference in the density of the inflammatory cells. Histological studies have shown that, after collagen cross-linking, the corneal stroma of rabbits will appear as lattice-like edema, which affects corneal transparency [38], and these edemas usually take 4 to 6 weeks to gradually subside [39]. Therefore, at the end of this study, there was no difference in corneal opacity and neovascularization between the two groups, which needs to be clarified by subsequent long-term follow-up studies.

The sample size of this study was small, and the follow-up time was short; therefore, a larger sample size and longer follow-up time are needed for further verification. Moreover, this study only compared the efficacy differences of different CXL protocols on single fungal species, and the efficacy differences for other common pathogenic fungi of keratitis need to be studied further. At the end of the study, the IVCM and histological results showed that no fungal hyphae or spores were found in any of the rabbits. The fungal bioburden of the infected corneas was not examined, which is a better quantitative method for assessing treatment efficacy.

## 5. Conclusions

In this study, the efficacy of voriconazole combined with standard CXL (3 mW/cm^2^*∗*30 min) and voriconazole combined with accelerated CXL (9 mW/cm^2^*∗*10 min) was compared in an animal model of fungal keratitis infected with *Aspergillus*. It was found that voriconazole combined with accelerated CXL was generally superior to voriconazole combined with standard CXL, which could control infection faster and promote ulcer healing. However, it is important to note that there is a risk of early deterioration of the ulcer with accelerated CXL. Therefore, when the ulcer is larger and deeper, it is necessary to pay attention to the safety of its application.

## Figures and Tables

**Table 1:** Corneal and corneal epithelium thickness between groups.

		Base line mean ± SD 95%CI	28 days after CXL mean ± SD 95%CI	*P*
Corneal epithelium thickness (*μ*m)	Accelerated CXL (*n*/*n*' = 12/12)	45.36 ± 2.24 43.94 to 46.78	48.13 ± 3.55 45.87 to 50.39	0.0323
	Standard CXL (*n*/*n*' = 12/11)	45.48 ± 2.17 44.10 to 46.86	50.67 ± 4.02 47.97 to 53.37	0.0008
	*P*	0.8952	0.1225	
Corneal thickness (*μ*m)	Accelerated CXL (*n*/*n*' = 12/12)	383.22 ± 27.35 365.83 to 400.62	537.68 ± 39.32 512.59 to 562.63	<0.0001
	Standard CXL (*n*/*n*' = 12/11)	381.80 ± 31.24 362.04 to 401.64	568.24 ± 44.59 538.23 to 598.18	<0.0001
	*P*	0.9068	0.0949	

CXL, corneal collagen cross-linking. The test used for statistical analysis among groups was *t*-test and between groups was one-way ANOVA. *n*/*n*', number of rabbits at base line/number of rabbits with non-healing excluded at 28 days after CXL.

## Data Availability

The data that support the findings of this study are available from the corresponding author upon reasonable request.

## References

[1] Wollensak G., Spoerl E., Seiler T. (2003). Riboflavin/ultraviolet-a-induced collagen crosslinking for the treatment of keratoconus. *American Journal of Ophthalmology*.

[2] Wollensak G. (2006). Crosslinking treatment of progressive keratoconus: new hope. *Current Opinion in Ophthalmology*.

[3] Frei R., Breitbach A. S., Blackwell H. E. (2012). 2-Aminobenzimidazole derivatives strongly inhibit and disperse *Pseudomonas aeruginosa* biofilms. *Angewandte Chemie*.

[4] Celik U. H., Alagoz N., Yildirim Y. (2012). Accelerated corneal crosslinking concurrent with laser in situ keratomileusis. *Journal of Cataract & Refractive Surgery*.

[5] Ting D. S. J., Rana-Rahman R., Chen Y. (2019). Effectiveness and safety of accelerated (9 mW/cm (2)) corneal collagen cross-linking for progressive keratoconus: a 24-month follow-up. *Eye*.

[6] Kamaev P., Friedman M. D., Sherr E., Muller D. (2012). Photochemical kinetics of corneal cross-linking with riboflavin. *Investigative Ophthalmology & Visual Science*.

[7] Elbaz U., Shen C., Lichtinger A. (2015). Accelerated versus standard corneal collagen crosslinking combined with same day phototherapeutic keratectomy and single intrastromal ring segment implantation for keratoconus. *British Journal of Ophthalmology*.

[8] Cinar Y., Cingu A. K., Turkcu F. M. (2014). Comparison of accelerated and conventional corneal collagen cross-linking for progressive keratoconus. *Cutaneous and Ocular Toxicology*.

[9] Roizenblatt R., Chai D., Gaster R. N., Farid M., Herekar S., Jester J. V. (2010). Comparison study of ultraviolet A irradiance of 3mW/cm2 versus 9mW/cm2 with riboflavin on corneal collagen cross-linking efficacy in rabbit eyes. *Investigative Ophthalmology & Visual Science*.

[10] Wen D., Li Q., Song B. (2018). Comparison of standard versus accelerated corneal collagen cross-linking for keratoconus: a meta-analysis. *Investigative Ophthalmology & Visual Science*.

[11] Manikandan P., Abdel-Hadi A., Randhir Babu Singh Y. (2019). Fungal keratitis: epidemiology, rapid detection, and antifungal susceptibilities of Fusarium and Aspergillus isolates from corneal scrapings. *BioMed Research International*.

[12] Ting D. S. J., Galal M., Kulkarni B. (2021). Clinical characteristics and outcomes of fungal keratitis in the United Kingdom 2011-2020: A 10-year study. *Journal of Fungi*.

[13] Khor W. B., Prajna V. N., Garg P. (2018). The asia cornea society infectious keratitis study: a prospective multicenter study of infectious keratitis in asia. *American Journal of Ophthalmology*.

[14] Mahmoudi S., Masoomi A., Ahmadikia K. (2018). Fungal keratitis: an overview of clinical and laboratory aspects. *Mycoses*.

[15] Ansari Z., Miller D., Galor A. (2013). Current thoughts in fungal keratitis: diagnosis and treatment. *Curr Fungal Infect Rep*.

[16] Wu J., Zhang W. S., Zhao J., Zhou H. Y. (2016). Review of clinical and basic approaches of fungal keratitis. *International Journal of Ophthalmology*.

[17] Iseli H. P., Thiel M. A., Hafezi F., Kampmeier J., Seiler T. (2008). Ultraviolet A/riboflavin corneal cross-linking for infectious keratitis associated with corneal melts. *Cornea*.

[18] Anwar H. M., El-Danasoury A. M., Hashem A. N. (2011). Corneal collagen crosslinking in the treatment of infectious keratitis. *Clinical Ophthalmology*.

[19] Bilgihan K., Kalkanci A., Ozdemir H. B. (2016). Evaluation of antifungal efficacy of 0.1% and 0.25% riboflavin with UVA: a comparative in vitro study. *Current Eye Research*.

[20] Chan T. C. Y., Lau T. W. S., Lee J. W. Y., Wong I. Y. H., Jhanji V., Wong R. L. M. (2015). Corneal collagen cross-linking for infectious keratitis: an update of clinical studies. *Acta Ophthalmologica*.

[21] Wei A., Wang K., Wang Y., Gong L., Xu J., Shao T. (2019). Evaluation of corneal cross-linking as adjuvant therapy for the management of fungal keratitis. *Graefes Archive for Clinical and Experimental Ophthalmology*.

[22] Papaioannou L., Miligkos M., Papathanassiou M. (2016). Corneal collagen cross-linking for infectious keratitis: a systematic review and meta-analysis. *Cornea*.

[23] Ting D. S. J., Henein C., Said D. G., Dua H. S. (2019). Photoactivated chromophore for infectious keratitis—corneal cross-linking (PACK-CXL): a systematic review and meta-analysis. *Ocular Surface*.

[24] Prajna N. V., Radhakrishnan N., Lalitha P. (2020). Cross-linking-assisted infection reduction: a randomized clinical trial evaluating the effect of adjuvant cross-linking on outcomes in fungal keratitis. *Ophthalmology*.

[25] Ting D. S. J., Henein C., Said D. G., Dua H. S. (2020). Re: Prajna et al.: cross-Linking-Assisted Infection reduction (CLAIR): a randomized clinical trial evaluating the effect of adjuvant cross-linking on outcomes in fungal keratitis. *Ophthalmology*.

[26] Schreiber W., Olbrisch A., Vorwerk C. K., Konig W., Behrens-Baumann W. (2003). Combined topical fluconazole and corticosteroid treatment for experimental Candida albicans keratomycosis. *Investigative Ophthalmology & Visual Science*.

[27] Li Z., Jhanji V., Tao X., Yu H., Chen W., Mu G. (2013). Riboflavin/ultravoilet light-mediated crosslinking for fungal keratitis. *British Journal of Ophthalmology*.

[28] Zhu Z., Zhang H., Yue J., Liu S., Li Z., Wang L. (2018). Antimicrobial efficacy of corneal cross-linking in vitro and in vivo for Fusarium solani: a potential new treatment for fungal keratitis. *BMC Ophthalmology*.

[29] Ozdemir H. B., Kalkanci A., Bilgihan K. (2019). Comparison of corneal collagen cross-linking (PACK-CXL) and voriconazole treatments in experimental fungal keratitis. *Acta Ophthalmologica*.

[30] Richoz O., Kling S., Hoogewoud F. (2014). Antibacterial efficacy of accelerated photoactivated chromophore for keratitis–corneal collagen cross-linking (PACK-CXL). *Journal of Refractive Surgery*.

[31] Herekar S. V. (2009). Method for equi-dosed time fractionated pulsed UVA irradiation of collagen/riboflavin mixtures for ocular structural augmentation. *Google Patents*.

[32] Holopainen J. M., Krootila K. (2011). Transient corneal thinning in eyes undergoing corneal cross-linking. *American Journal of Ophthalmology*.

[33] Kalkanci A., Yesilirmak N., Ozdemir H. B. (2018). Impact of iontophoresis and PACK-CXL corneal concentrations of antifungals in an in vivo model. *Cornea*.

[34] Stewart J. M., Lee O. T., Wong F. F., Schultz D. S., Lamy R. (2011). Cross-linking with ultraviolet-a and riboflavin reduces corneal permeability. *Investigative Ophthalmology & Visual Science*.

[35] Wollensak G., Spoerl E., Wilsch M., Seiler T. (2004). Keratocyte apoptosis after corneal collagen cross-linking using riboflavin/UVA treatment. *Cornea*.

[36] Ghanem R. C., Netto M. V., Ghanem V. C., Santhiago M. R., Wilson S. E. (2012). Peripheral sterile corneal ring infiltrate after riboflavin–UVA collagen cross-linking in keratoconus. *Cornea*.

[37] Chanbour W., Mokdad I., Mouhajer A., Jarade E. (2019). Late-onset sterile peripheral ulcerative keratitis post-corneal collagen crosslinking. *Cornea*.

[38] Wollensak G., Herbst H. (2010). Significance of the lacunar hydration pattern after corneal cross linking. *Cornea*.

[39] Croxatto J. O., Tytiun A. E., Argento C. J. (2010). Sequential in vivo confocal microscopy study of corneal wound healing after cross-linking in patients with keratoconus. *Journal of Refractive Surgery*.

